# The Wrist Circumference-to-Body Mass Index Ratio for Preprocedural Risk Stratification of Radial Artery Spasm in Transradial Coronary Angiography and Percutaneous Coronary Intervention

**DOI:** 10.3390/diagnostics16040643

**Published:** 2026-02-23

**Authors:** Ahmet Can Çakmak, Betül Sarıbıyık Çakmak, Muhammed Necati Murat Aksoy

**Affiliations:** 1 Department of Cardiology, Sakarya Training and Research Hospital, 54100 Sakarya, Türkiye; 2 Department of Cardiology, Yenikent State Hospital, 54100 Sakarya, Türkiye; betul.saribiyik@gmail.com; 3 Department of Cardiology, Sakarya University Faculty of Medicine, 54050 Sakarya, Türkiye; draxoy@gmail.com

**Keywords:** radial artery spasm, transradial access, coronary angiography, wrist circumference, body mass index, anthropometry, vascular complications

## Abstract

**Objectives**: Radial artery spasm (RAS) is a common complication of transradial coronary angiography that may adversely affect procedural success and patient comfort. This study aimed to evaluate clinical, procedural, and anthropometric factors associated with RAS in patients undergoing elective transradial coronary angiography, with a particular focus on the wrist circumference-to-body mass index (WC/BMI) ratio as a novel predictor. **Methods**: A total of 466 patients who underwent elective coronary angiography via the right radial artery between January 2024 and December 2024 were included. All procedures were performed using a 6 Fr introducer sheath according to a standardized protocol. Radial artery spasm was clinically defined as operator resistance during catheter manipulation accompanied by patient-reported pain or marked discomfort in the accessed arm. Wrist circumference and body mass index were measured before the procedure, and the WC/BMI ratio was calculated. Radial artery diameter was assessed using ultrasonography. Variables associated with RAS were evaluated using univariable and multivariable logistic regression analyses. Due to collinearity between WC/BMI and radial artery diameter, two separate multivariable models were constructed. Discriminative performance was assessed using receiver operating characteristic (ROC) curve analysis. **Results**: Radial artery spasm occurred in 51 patients (10.9%). Patients who developed RAS had significantly lower WC/BMI ratios and smaller radial artery diameters compared with those without spasm (both *p* ≤ 0.001). In multivariable analysis, a lower WC/BMI ratio was independently associated with an increased risk of RAS (odds ratio [OR] 0.51 per 0.1-unit increase; 95% confidence interval [CI] 0.34–0.78; *p* = 0.002). Similarly, smaller radial artery diameter remained an independent predictor of RAS (OR 0.83 per 0.1 mm increase; 95% CI 0.75–0.92; *p* < 0.001). The area under the curve (AUC) was 0.651 for WC/BMI and 0.636 for radial artery diameter. The combined model demonstrated improved discriminative ability (AUC 0.713). **Conclusions**: The WC/BMI ratio is a simple, practical, and readily obtainable anthropometric parameter that can predict the risk of radial artery spasm before transradial coronary angiography. When combined with radial artery diameter, it provides improved discrimination for identifying patients at higher risk of RAS.

## 1. Introduction

Radial artery access (TRA) has become the preferred vascular approach for diagnostic coronary angiography and percutaneous coronary interventions because it is associated with a lower risk of bleeding, earlier mobilization, and improved patient comfort compared with femoral access [1,2]. Radial artery spasm (RAS) is a common complication of TRA in both elective and emergency coronary procedures and may lead to severe pain and procedural failure [3,4].

The reported incidence of radial artery spasm varies between 4% and 20%, largely due to the lack of a standardized definition for this complication, with rates exceeding 50% reported in some studies [3,5]. The pathophysiology of RAS is thought to be related to the dense distribution of adrenoreceptors within the radial artery wall, which increases vasoreactivity and leads to smooth muscle vasoconstriction when triggered by catheter manipulation, vessel wall stretch, and local trauma [3].

Clinically, radial artery spasm (RAS) is defined as pain or discomfort perceived by the patient in the forearm during catheter insertion, manipulation, or withdrawal of the introducer sheath [6]. Assessment of RAS is predominantly based on the operator’s clinical observation; however, various angiographic and subjective criteria, such as luminal narrowing greater than 25%, 30%, 50%, or 75%, have also been used to define RAS [7,8,9,10]. In addition, automated sheath pullback devices have been employed as alternative methods for the assessment of RAS [5].

For the prevention of RAS, intra-arterial vasodilator cocktails containing agents such as verapamil, nitroglycerin, nitrates, or nicorandil, with or without heparin, are widely used. Numerous risk factors for the development of RAS have been identified, including emergency procedures, anatomical variations of the radial artery, smaller radial artery diameter, shorter stature, lower body mass index, female sex, dyslipidemia, patient anxiety, the number of puncture attempts, excessive catheter manipulation, procedure duration, the number of catheters used, and higher contrast media volume [3,4].

However, a substantial proportion of the parameters used to predict radial artery spasm are based either on factors that arise during the procedure or on anthropometric measurements evaluated in isolation. Although body mass index is a systemic measure, it does not adequately reflect distal extremity anatomy. Considering the superficial location of the radial artery and its close anatomical relationship with wrist circumference, normalizing wrist circumference to body mass index may serve as a practical anthropometric parameter that could indirectly represent patient-specific distal arterial structure. The wrist circumference-to-body mass index (WC/BMI) ratio may reflect the relationship between distal extremity anatomy and overall body habitus and could serve as an indirect marker of the radial artery’s vulnerability to mechanical irritation. From this perspective, WC/BMI is not proposed as a diagnostic marker, but rather as a potential preprocedural screening parameter to identify patients who may benefit from preventive strategies before transradial coronary angiography. Therefore, the present study aimed to evaluate the association between WC/BMI and the development of radial artery spasm.

## 2. Materials and Methods

### 2.1. Patient Selection and Methods

Between January 2024 and December 2024, a total of 500 patients aged 18 years or older who were scheduled to undergo elective coronary angiography (CAG) via the right radial artery at Sakarya Training and Research Hospital were initially enrolled in the study. Patients with known radial or ulnar artery malformations, radial or ulnar artery vaso-occlusive disease, a history of transradial angiography within the preceding 3 months, a history of coronary artery bypass surgery, active malignancy, advanced chronic kidney disease (e.g., estimated glomerular filtration rate [eGFR] < 30 mL/min/1.73 m^2^ or dialysis), or known chronic inflammatory diseases (such as rheumatoid arthritis or systemic lupus erythematosus) were excluded. In addition, 34 patients were not included in the analysis because they had anxiety requiring sedation during the procedure, required conversion to a 7 Fr sheath during the procedure (e.g., for complex lesions), or had a vascular access route other than the transradial approach selected by the operator. The final analysis was performed on a total of 466 patients. Baseline demographic characteristics and clinical data of all patients were recorded. Coronary artery disease (CAD) was defined as ≥50% luminal stenosis in at least one major epicardial coronary artery documented by coronary angiography, or a history of percutaneous coronary intervention or myocardial infarction. Chronic kidney disease (CKD) was defined as an estimated glomerular filtration rate (eGFR) of <60 mL/min/1.73 m^2^ persisting for at least 3 months.

Interventional procedures were performed at a high-volume center with more than 100 transradial coronary procedures per week by three experienced interventional cardiologists, each of whom had performed more than 1000 transradial procedures. The coronary angiography laboratory was air-conditioned, and room temperature was maintained between 20 and 25 °C. Before the procedure, vital signs, including blood pressure and heart rate, were measured and recorded. All patients received local anesthesia with 2–3 mL of 2% lidocaine prior to arterial puncture. Radial artery puncture was performed using a 20-gauge needle. In all cases, the same brand of 6 Fr introducer sheath (Shun Guider Introducer Sets, Shunmei Medical Co., Ltd., Shenzhen, China) was used. The introducer sheath used in this study was non-hydrophilic coated. All procedures were initially started using a 6 Fr sheath. Patients in whom the operator decided to convert to a 7 Fr sheath during the procedure (e.g., for left main or complex bifurcation lesions) were excluded from the study. After sheath insertion, an intra-arterial cocktail containing 100 µg nitroglycerin and 5000 IU heparin was administered through the sheath in all patients. The cocktail was administered as a slow intra-arterial injection over approximately 10–15 s to minimize local endothelial irritation.

For catheter navigation, a 0.038-inch guidewire was routinely used, and a 0.035-inch hydrophilic guidewire was used when necessary. In all cases, a 6 Fr introducer sheath was used in accordance with the institutional standard practice. Initial cannulation of the right and left coronary arteries was routinely performed using a 5 Fr Tiger II diagnostic catheter. In cases where selective cannulation could not be achieved with the Tiger II catheter or when percutaneous coronary intervention was planned, the procedure was continued at the operator’s discretion using 6 Fr diagnostic (Judkins or Amplatz) and/or 6 Fr guiding (Judkins, Extra Back-Up, or Amplatz) catheters, provided that the procedure was completed with a 6 Fr sheath. Procedure duration was defined as the time from radial artery puncture to removal of the intra-arterial sheath.

### 2.2. Anthropometric Measurements

Preprocedural wrist circumference was measured while the patient was in a seated position, with the hand relaxed and in a neutral position, using a non-elastic measuring tape without applying pressure to the skin, just proximal to the radial styloid process. Patients’ height and weight were measured, and body mass index (BMI) was calculated. The wrist circumference-to-BMI ratio (WC/BMI) was calculated for all patients before the procedure and recorded. Demographic data and medical history were obtained from patient self-report and hospital records.

### 2.3. Ultrasonographic Assessment of the Radial Artery

Radial artery ultrasonographic measurements were performed before the procedure using a Vivid S70N B-mode ultrasonography system equipped with a 7 MHz linear probe, by a single experienced physician, and were recorded. Measurements were obtained 1–2 cm proximal to the radial styloid process. Intra-observer reproducibility of radial artery diameter measurements was assessed in repeated measurements, yielding a coefficient of variation of 4%.

### 2.4. Definition of Radial Artery Spasm and Procedural Data

The definition of radial artery spasm was based on predefined standardized criteria agreed upon by all operators. Radial artery spasm was clinically defined as operator resistance during advancement or withdrawal of the catheter during manipulation, accompanied by patient-reported pain or marked discomfort in the accessed arm. Assessment of radial artery spasm was performed in real time during the procedure and was confirmed by the operator after completion of the procedure. Procedural data, including the presence of radial artery spasm, procedure duration, contrast volume used, and the number of catheters utilized, were recorded after the procedure.

### 2.5. Ethics Committee Approval and Informed Consent

This study was reviewed and approved by the Clinical Research Ethics Committee of Sakarya University Faculty of Medicine, Sakarya Training and Research Hospital (Approval No: 16214662/050.01.04/48773-125). The study was conducted in accordance with the principles of the Declaration of Helsinki. Written informed consent was obtained from all participants prior to enrollment.

### 2.6. Statistical Analyses

Statistical analyses were performed using SPSS software (IBM SPSS Statistics, version 21.0; IBM Corp., Armonk, NY, USA). Continuous variables were first assessed for normality using the Shapiro–Wilk test. Normally distributed variables are expressed as mean ± standard deviation and compared using Student’s *t*-test, whereas non-normally distributed variables are presented as median (interquartile range, IQR) and compared using the Mann–Whitney U test. Categorical variables are expressed as counts and percentages and compared using the chi-square test or Fisher’s exact test, as appropriate.

Univariable analyses were conducted to identify variables associated with the development of radial artery spasm. Variables with a *p* value < 0.10 in univariable analyses and those considered clinically relevant were considered candidates for multivariable logistic regression analysis. Multicollinearity among candidate variables was assessed using variance inflation factor (VIF), with a VIF value > 5 considered indicative of significant multicollinearity. Due to significant collinearity between the wrist circumference/body mass index ratio (WC/BMI) and radial artery diameter, these variables were not included in the same multivariable model. Therefore, two separate multivariable logistic regression models were constructed: an anthropometric index model including WC/BMI and an anatomical model including radial artery diameter. For statistical modeling, radial artery diameter was scaled in 0.1 mm units (i.e., multiplied by 10) to improve interpretability of odds ratios. Results of multivariable analyses are reported as β coefficients (log-odds), odds ratios (ORs), and 95% confidence intervals (CIs). For improved interpretability, WC/BMI was scaled per 0.1-unit increase in the multivariable analysis. Variables not included in the multivariable models were excluded either because of lack of statistical significance in univariable analysis or due to potential collinearity with procedural complexity variables. Because escalation beyond the initial diagnostic step involved additional catheters and the use of 6 Fr diagnostic or guiding catheters, the number of catheters was treated as a surrogate marker of procedural complexity (and indirectly of larger catheter exposure); therefore, catheter size was not entered as a separate covariate to avoid redundancy and collinearity.

Accordingly, WC/BMI and radial artery diameter were evaluated in separate multivariable models for inference. However, both variables were included only in a prediction-only model to generate probabilities for the combined ROC analysis, and coefficients from this model were not interpreted as independent effects.

Receiver operating characteristic (ROC) curve analysis was performed to evaluate the discriminative ability of WC/BMI and radial artery diameter for predicting radial artery spasm. In addition, a combined ROC curve was generated using predicted probabilities obtained from a predictive model incorporating WC/BMI and radial artery diameter, with the aim of evaluating their joint discriminative performance. The optimal cut-off values were determined using the Youden index. The area under the curve (AUC), sensitivity, and specificity were calculated for each parameter. A two-sided *p* value < 0.05 was considered statistically significant for all analyses.

## 3. Results

A total of 466 patients undergoing elective coronary angiography via the right radial artery were included in the final analysis. Radial artery spasm occurred in 51 patients, yielding an overall spasm rate of 10.9%.

Baseline clinical, demographic, and anthropometric characteristics of patients with and without radial artery spasm are summarized in Table 1. Age, body weight, body mass index, systolic and diastolic blood pressure, heart rate, and the prevalence of cardiovascular comorbidities did not differ significantly between the two groups.

Female sex was significantly more frequent among patients who developed radial artery spasm compared with those without spasm (56.9% vs. 37.6%, *p* = 0.01). Patients with radial artery spasm were shorter in height (*p* = 0.004) and had a smaller wrist circumference (*p* = 0.01). In addition, both the wrist circumference/body mass index ratio (WC/BMI) and radial artery diameter were significantly lower in the spasm group compared with the non-spasm group (both *p* ≤ 0.001).

Procedural characteristics according to the presence of radial artery spasm are presented in Table 2. Multiple puncture was significantly more frequent in patients who developed radial artery spasm compared with those without spasm (51.0% vs. 20.7%, *p* < 0.001). Patients in the spasm group required a higher number of catheters, had longer procedure duration, and received a greater volume of contrast media than those without spasm.

Results of the multivariable logistic regression analyses are shown in Table 3. In both multivariable models, multiple puncture and number of catheters emerged as independent predictors of radial artery spasm.

In the anthropometric index model (Table 3), a lower WC/BMI (per 0.1-unit increase) was independently associated with a higher risk of radial artery spasm (OR 0.51, 95% CI 0.34–0.78, *p* = 0.002), whereas procedure duration did not remain an independent predictor after adjustment.

Multivariable logistic regression analysis shows independent predictors of radial artery spasm in the anthropometric index model. The wrist circumference/body mass index ratio (WC/BMI) was entered into the model scaled per 0.1-unit increase. Odds ratios (ORs) are presented with 95% confidence intervals (CIs).

In the anatomical model (Table 3), smaller radial artery diameter was independently associated with the occurrence of radial artery spasm (OR 0.83 per 0.1 mm increase, 95% CI 0.75–0.92, *p* < 0.001). Procedure duration showed a borderline association with radial artery spasm but did not reach statistical significance after adjustment (*p* = 0.082).

Multivariable logistic regression analysis shows independent factors associated with radial artery spasm in the anatomical model including radial artery diameter. Radial artery diameter was entered into the model per 0.1 mm increase; therefore, odds ratios (ORs) represent the effect per 0.1 mm increase. Odds ratios are presented with 95% confidence intervals (CIs).

**Figure 1:**
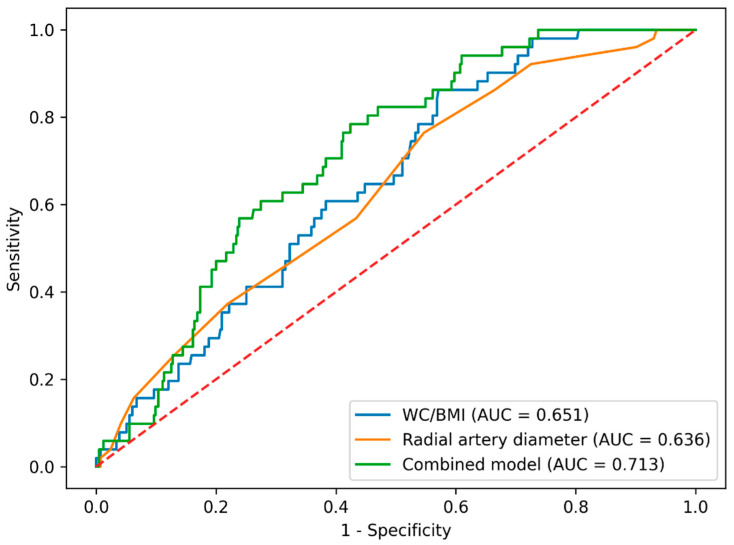
ROC curves for prediction of radial artery spasm. The red dotted line indicates the optimal cut-off value derived from the Youden index.

ROC curve analysis was performed to evaluate the discriminative ability of WC/BMI and radial artery diameter, as well as their combined model, for predicting radial artery spasm (Table 4, Figure 1). The area under the curve (AUC) for WC/BMI was 0.651, with an optimal cut-off value of ≤0.65, yielding a sensitivity of 86.3% and specificity of 42.9%. For radial artery diameter, the AUC was 0.636, and a cut-off value of ≤2.7 mm provided a sensitivity of 76.5% and specificity of 45.3%. The combined model demonstrated improved discriminative performance, with an AUC of 0.713; at the optimal cut-off value, sensitivity and specificity were 78.4% and 57.6%, respectively.

WC/BMI indicates wrist circumference-to-body mass index ratio. ROC analyses were performed to evaluate the discriminative ability of WC/BMI, radial artery diameter, and their combined model for predicting radial artery spasm. The combined ROC curve was generated using predicted probabilities obtained from a predictive model incorporating WC/BMI and radial artery diameter to assess their joint discriminative performance. The cut-off value for the combined model represents the predicted probability. Because the event rate of radial artery spasm was 10.9%, the optimal probability threshold may fall below 0.10 depending on the sensitivity–specificity trade-off.

## 4. Discussion

In this study, clinical, procedural, and anthropometric factors associated with the development of radial artery spasm (RAS) were evaluated in patients undergoing elective coronary angiography. The principal finding of the study is that the wrist circumference-to-body mass index ratio (WC/BMI) was significantly associated with the development of radial artery spasm, and that this association was more pronounced than when body mass index was evaluated alone. The fact that WC/BMI is a practical parameter that can be easily measured before the procedure and is clinically applicable makes this finding particularly noteworthy.

In our study, radial artery spasm was observed in 51 of the 466 patients analyzed, corresponding to an overall spasm rate of 10.9%. In a previous study conducted at our center involving 159 patients, the incidence of RAS was also reported as 10% [11]. These rates are consistent with the incidence of spasm reported in the literature following transradial procedures, which ranges between 4% and 20% [3,5]. However, the reported incidence of RAS largely depends on the subjective diagnostic criteria used across studies. The establishment of a standardized definition could facilitate future research in this field and allow for more meaningful comparisons between studies.

Radial artery spasm remains an important complication that directly affects the success of the transradial approach and patient comfort. Therefore, the ability to predict the development of radial artery spasm before the procedure is important, as it may prompt the operator to exercise greater caution and implement preventive measures. Risk factors for RAS reported in the literature include the number of puncture attempts, sheath and catheter size, prolonged procedure duration, increased catheter use, and higher contrast volume [12,13,14]. In the present study, a higher number of puncture attempts, longer procedure duration, greater catheter use, and higher contrast volume were also observed significantly more frequently in patients who developed RAS. Although procedure duration was significantly longer in patients with RAS in univariable analysis, it did not remain an independent predictor after adjustment, suggesting that its effect may have been mediated by procedural complexity, such as multiple puncture attempts and increased catheter use. These findings are consistent with previous studies and further emphasize the important role of procedural factors in the development of RAS. It should be acknowledged that intra-arterial heparin, which has an acidic pH, may itself represent a minor procedural trigger for radial artery spasm, particularly if administered rapidly. Although a standardized slow injection technique was used in all patients, this factor should be considered when interpreting our findings. Sheath coating is a known factor influencing the development of radial artery spasm. The use of a non-hydrophilic sheath in our study may have contributed to local mechanical irritation and should therefore be considered as a potential procedural factor when interpreting the results.

In our study, radial artery diameter was identified as a strong and independent predictor of radial artery spasm. Patients who developed RAS had a significantly smaller radial artery diameter compared with those who did not develop spasm. In multivariable logistic regression analysis, radial artery diameter remained independently and significantly associated with the development of RAS after adjustment for procedural and clinical covariates. These findings are in line with previous studies reporting that a smaller radial artery size increases the risk of spasm through a higher sheath-to-artery ratio, increased wall friction, and greater mechanical irritation [3,15]. In addition, the small caliber of the radial artery and its regulation predominantly via α_1_-adrenergic receptors contribute to increased vasoreactivity, rendering the vessel more susceptible to vasospasm in response to trauma [16,17,18].

In our study, female sex was observed to be associated with the development of radial artery spasm, a finding that is consistent with the existing literature. Several studies have reported female sex as an independent predictor of radial artery spasm [6,8,19]. However, in our multivariable analyses, female sex did not remain an independent predictor, suggesting that this association may be largely explained by differences in body size and, in particular, arterial diameter. Possible reasons why female sex has been reported as an important risk factor for spasm include differences in overall body size and arterial caliber, as well as a higher prevalence and greater intensity of procedure-related anxiety in women, which may further increase the risk of spasm development [20].

In this study, shorter height was found to be statistically significantly associated with the development of radial artery spasm. This finding is supported by previous studies in the literature [21,22]. Notably, a predictive score proposed by Giannopoulos et al. assigns substantial weight to a height of less than 170 cm as a risk factor for radial artery spasm [23]. In addition, wrist circumference was also found to be statistically significant in relation to RAS in our study. A previous study similarly demonstrated a significant association between wrist circumference and the development of radial artery spasm [24]. Furthermore, several studies have reported a significant relationship between wrist circumference and radial artery size, supporting the biological plausibility of this association [25,26].

Age, body weight, heart rate, diabetes mellitus, hypertension, peripheral artery disease, coronary artery disease, and smoking status were not found to be significantly associated with radial artery spasm in our study. There are studies in the literature that support these findings [14,27]. The lack of a significant association between radial artery spasm and classical cardiovascular risk factors such as smoking, hypertension, and diabetes suggests that RAS may be more closely related to local and mechanical factors rather than systemic atherosclerotic burden. However, in contrast to our findings, some studies in the literature have identified peripheral artery disease, diabetes, and smoking as risk factors for RAS [22,23,28]. Therefore, larger studies are warranted to further clarify these associations.

One of the distinctive aspects of our study is the evaluation of a novel anthropometric parameter, WC/BMI, for predicting radial artery spasm. Although body mass index is a systemic measure, it may not adequately reflect distal extremity anatomy or the relationship between the radial artery and surrounding tissues. Indeed, in our study, BMI alone was not significantly associated with RAS, a finding that is consistent with previous reports demonstrating no significant relationship between BMI and radial artery spasm [27,29]. In contrast, normalizing wrist circumference to BMI provided a more comprehensive representation of patient-specific distal arterial structure, and WC/BMI emerged as an independent predictor of RAS risk. This finding suggests that patients with a lower BMI but a relatively larger wrist circumference may have a lower risk of developing radial artery spasm.

ROC analysis demonstrated that WC/BMI showed a moderate discriminative performance for predicting radial artery spasm, with high sensitivity but limited specificity. Although the discriminative ability of WC/BMI alone was moderate (AUC: 0.651), its primary clinical value lies in its high sensitivity and ease of bedside application. From a clinical perspective, this finding suggests that WC/BMI should not be interpreted as a standalone diagnostic marker, but rather as a simple and rapid preprocedural screening tool to identify patients at higher risk for radial artery spasm. In addition, the combined model incorporating both WC/BMI and radial artery diameter exhibited better discriminative performance than either parameter alone, supporting the concept that radial artery spasm develops as a result of the combined effects of anthropometric and anatomical factors rather than a single measurement. In this context, early identification of patients at high risk for RAS prior to transradial procedures may allow optimization of vasodilator strategies and, when necessary, planning of alternative vascular access options, making this combined approach clinically useful.

## 5. Limitations

This study was conducted at a single center and included a relatively limited number of patients. Although efforts were made to standardize the diagnosis of radial artery spasm, it was based on clinical assessment. The absence of angiographic standardization or the use of automated pullback devices for a less operator-dependent diagnosis represents a limitation of the study. In addition, although patients with agitation severe enough to require sedation were excluded, no standardized questionnaire was used to assess agitation levels in the remaining patients, which constitutes another limitation. Furthermore, although patients with known radial artery malformations were excluded, the presence of angiographically unrecognized radial artery malformations in the included patients was not systematically assessed, representing an additional limitation.

## 6. Conclusions

In conclusion, this study demonstrates that WC/BMI is a practical anthropometric parameter that is easily measurable before the procedure and is associated with the development of radial artery spasm. Because these measurements are obtained preprocedurally and provide insight into the risk of RAS before the procedure begins, they have distinct importance in addition to procedural factors associated with RAS development during the procedure, such as the number of catheters used and multiple puncture attempts. Incorporating WC/BMI alongside existing clinical and procedural risk factors may be beneficial for patient selection and for predicting the risk of complications in transradial interventions.

## Figures and Tables

**Table 1:** Clinical, Demographic, and Anthropometric Characteristics of Patients With and Without Radial Artery Spasm.

Characteristics	No RAS (*n* = 415)	RAS (*n* = 51)	*p* Value
Age, years	59.12 ± 9.93	59.06 ± 10.12	0.97
Female sex, *n* (%)	156 (37.6)	29 (56.9)	0.01
Height, cm	167.07 ± 8.50	163.49 ± 7.99	0.004
Weight, kg	80 (72–92)	80 (74.5–89.5)	0.88
Body mass index, kg/m^2^	29.26 (26.32–32.50)	29.40 (27.46–34.69)	0.14
Wrist circumference, cm	18 (17.5–19)	17.5 (16.5–18.5)	0.01
WC/BMI ratio, cm/(kg/m^2^)	0.62 (0.57–0.69)	0.58 (0.54–0.63)	<0.001
Radial artery diameter, mm	2.7 (2.5–3.0)	2.6 (2.35–2.7)	0.001
Hypertension, *n* (%)	258 (62.2)	33 (64.7)	0.84
Diabetes mellitus, *n* (%)	150 (36.1)	20 (39.2)	0.78
Coronary artery disease, *n* (%)	177 (42.7)	18 (35.3)	0.37
Peripheral artery disease, *n* (%)	3 (0.7)	0 (0.0)	1
Smoking, *n* (%)	258 (62.2)	28 (54.9)	0.36
Chronic kidney disease, *n* (%)	16 (3.9)	0 (0.0)	0.24
Heart rate, bpm	79 (70–88)	81 (75–89)	0.14
Systolic BP, mmHg	130 (120–140)	125 (120–140)	0.91
Diastolic BP, mmHg	80 (70–80)	80 (70–90)	0.79

Continuous variables are presented as mean ± SD or median (IQR), depending on data distribution. WC/BMI = wrist circumference-to-body mass index ratio.

**Table 2:** Procedural Characteristics.

Characteristics	No RAS (*n* = 415)	RAS (*n* = 51)	*p* Value
Procedure duration (min)	8 (5–15)	20 (14–27)	<0.001
Contrast volume (mL)	50 (30–90)	100 (60–150)	<0.001
Number of catheters	1 (1–1)	2 (1–2)	<0.001
Multiple puncture attempts, *n* (%)	86 (20.7)	26 (51.0)	<0.001

Data are presented as median (IQR) or n (%), as appropriate.

**Table 3:** Multivariable logistic regression analysis.

**(A) Multivariable Logistic Regression Analysis (Anthropometric Index Model)**				
**Variable**	**β (Log-Odds)**	**Odds Ratio (OR)**	**95% Confidence Interval**	***p*** **Value**
WC/BMI (per 0.1 increase)	−0.67	0.51	0.34–0.78	0.002
Multiple puncture attempts	1.28	3.61	1.9–6.86	<0.001
Procedure duration (min)	0.02	1.02	0.99–1.04	0.15
Number of catheters used	0.88	2.41	1.5–3.88	<0.001
**(B) Multivariable Logistic Regression Analysis (Anatomical Model)**				
Radial artery diameter (per 0.1 mm increase)	−0.19	0.83	0.75–0.92	<0.001
Multiple puncture attempts	1.33	3.78	1.98–7.25	<0.001
Procedure duration (min)	0.02	1.02	1.00–1.04	0.08
Number of catheters used	0.98	2.66	1.66–4.27	<0.001

**Table 4:** Receiver Operating Characteristic Curve Analysis.

Model	AUC	95% CI	Optimal Cut-Off	Sensitivity (%)	Specificity (%)
WC/BMI	0.651	0.582–0.721	≤0.65	86.3	42.9
Radial artery diameter (mm)	0.636	0.557–0.712	≤2.7	76.5	45.3
Combined model (WC/BMI + radial artery diameter)	0.713	0.649–0.775	≥0.097 *	78.4	57.6

* Cut-off value represents the predicted probability derived from the combined model.

## Data Availability

The data presented in this study are not publicly available due to privacy and ethical restrictions but are available from the corresponding author upon reasonable request.
